# Changes in the Position of Anatomical Points, Cranio-Cervical Posture, and Nasopharyngeal Airspace Dimensions in Complete Denture Wearers—A Cephalometric Pilot Study

**DOI:** 10.3390/dj13080335

**Published:** 2025-07-22

**Authors:** Andrea Maria Chisnoiu, Mihaela Hedeșiu, Oana Chira, Iris Bara, Simona Iacob, Andreea Kui, Smaranda Buduru, Mihaela Păstrav, Mirela Fluerașu, Radu Chisnoiu

**Affiliations:** 1 Department of Prosthetic Dentistry and Dental Materials, Faculty of Dental Medicine, “Iuliu Hațieganu” University of Medicine and Pharmacy, 400012 Cluj-Napoca, Romania; maria.chisnoiu@umfcluj.ro (A.M.C.); iacob.simona@umfcluj.ro (S.I.); dana.buduru@umfcluj.ro (S.B.); fluerasu.mirela@umfcluj.ro (M.F.); 2 Department of Maxillofacial Surgery and Radiology, Faculty of Dental Medicine, “Iuliu Hațieganu” University of Medicine and Pharmacy, 400012 Cluj-Napoca, Romania; mhedesiu@umfcluj.ro; 3 Cluj County Emergency Clinical Hospital, 400347 Cluj-Napoca, Romania; sirb.oana.maria@eleran.umfcluj.ro (O.C.); iris.bara@elearn.umfcluj.ro (I.B.); 4 Department of Conservative Dentistry, Faculty of Dental Medicine, “Iuliu Hațieganu” University of Medicine and Pharmacy, 400012 Cluj-Napoca, Romania; mihaela.pastrav@umfcluj.ro (M.P.); marcel.chisnoiu@umfcluj.ro (R.C.)

**Keywords:** complete denture, cephalometry, posture

## Abstract

**Objectives**: The objective of this study was to evaluate changes in anatomical point position, cranio-cervical posture, and respiratory dimensions following conventional bimaxillary total prosthetic rehabilitation. **Methods:** A prospective, longitudinal, observational, analytical study was conducted on 12 patients, aged 55 to 75 years, at the Department of Dental Prosthetics at the University of Medicine and Pharmacy in Cluj-Napoca. All patients had complete bimaxillary edentulism and received removable dentures as treatment. Clinical and cephalometric analyses were performed before and after prosthetic treatment to compare changes. The cephalometric analysis was based on the guidelines of Tweed and Rocabado for evaluation. Quantitative data were described using the mean and standard deviation for normal distribution and represented by bar graphs with error bars. A paired samples t-test was used to determine differences between groups, with a significance threshold of 0.05 for the bilateral *p*-value. **Results**: When analyzing changes in cranial base inclination, the corresponding angles exhibited an increase, indicating cephalic extension. A statistically significant difference in the anteroposterior diameter of the oropharyngeal lumen with and without bimaxillary complete dentures was identified (*p* < 0.05). For hyperdivergent patients, modifications in the position of anatomical features on cephalometry slightly reduced the VDO and had a slight compensatory effect on skeletal typology. In contrast, for hypodivergent patients, modifications to the position of anatomical landmarks also had a compensatory effect on skeletal typology, increasing the VDO. **Conclusion**: Changes in the position of anatomical features on cephalometry generally have a compensatory effect on skeletal typology after complete denture placement. Complete prosthetic treatment with removable dentures can significantly influence respiratory function by reducing the oropharyngeal lumen and body posture by cephalic extension and attenuation of the lordotic curvature of the cervical spine.

## 1. Introduction

Population aging is a global trend. According to the United Nations (UN), the proportion of elderly individuals—those over the age of 60—is projected to increase, as detailed in a report by the UN Population Fund India. A common consequence of this trend is edentulism, or complete tooth loss. This irreversible and debilitating condition is often referred to as the “ultimate marker of disease burden for oral health” [1]. Despite a decline in the prevalence of complete tooth loss over the past decade, edentulism remains a significant global issue, particularly among the elderly. It is associated with physiological bone resorption and changes in muscles and soft tissues. It can also lead to a reduced range of motion in adjacent joints and alterations in head posture [2]

Recent epidemiological studies have provided updated insights into the global and regional prevalence of complete edentulism. In 2021, it was reported that approximately 353 million people were edentulous worldwide, with an age-standardized prevalence rate of around 4.11%. Notably, Latin America and the Caribbean exhibited the highest regional prevalence at approximately 7.39% [3]. Furthermore, a three-decade analysis (1990–2021) revealed substantial disparities in edentulism prevalence and disability-adjusted life years (DALYs) across regions, projecting ongoing trends until 2040. These findings highlight the ongoing public health impact of edentulism, particularly in regions with lower sociodemographic indices. [4].

In Romania, the prevalence of edentulism among individuals aged 20 years and older was estimated at 8.3% in 2019, according to the World Health Organization’s Oral Health Country Profile [5]. Furthermore, a study focusing on young adults in Iași, Romania, reported a decrease in edentulism prevalence from 43% in 2010 to 11% in 2014, highlighting the impact of targeted dental health initiatives [6]. These findings emphasize the need for continued public health efforts to address edentulism both globally and within Romania.

The natural head position is maintained by balancing the cranio-cervical bones, myofascial structures, and dental occlusion [7]. Loss of dental occlusal relationships can alter the skull’s orientation relative to the neck. These changes in head posture may disrupt neuromuscular articulations and vertebral alignment. They may also affect normal functions, such as mastication, phonation, and respiration. Additionally, they may affect the body’s center of gravity and potentially lead to postural imbalance [8,9].

Research suggests that rehabilitation with complete dentures can improve mastication, aesthetics, phonetics, head posture, and body balance. Manni et al. [9] found that dentures enhanced stability in individuals with mild-to-moderate dementia. Studies by Watanabe et al. [10] and Fujinami et al. [11] demonstrated that wearing complete dentures increases gait velocity and improves body balance. Okubo et al. [12] observed that complete dentures affect patient stability under static and dynamic conditions.

In recent years, significant progress has been made in the field of dental prosthetics to improve the quality of life for edentulous patients, particularly those requiring bimaxillary total prosthetic rehabilitation. Despite these advancements, the comprehensive impact of such treatments on anatomical points, cranio-cervical posture, and respiratory dimensions remains underexplored. This study addresses the gap by raising the following question: How does the use of complete dentures influence anatomical landmarks, cranio-cervical posture, and respiratory dimensions? This study aims to elucidate the complex changes that follow conventional bimaxillary prosthetic rehabilitation.

This study aimed to compare changes in anatomical point position, cranio-cervical posture, and respiratory dimensions following conventional bimaxillary total prosthetic rehabilitation. The null hypothesis proposed a direct link between complete denture usage and cranio-cervical posture and respiratory dimensions.

## 2. Methods

### 2.1. Study Design

This prospective longitudinal observational analytical study was conducted in accordance with the Declaration of Helsinki. Ethical approval was granted by the Institutional Review Board of the “Iuliu Hațieganu” University of Medicine and Pharmacy Cluj-Napoca (protocol code AVZ 8/6.01.2023).

### 2.2. Inclusion and Exclusion Criteria

Participants included adults aged 55 to 75 years presenting with complete bimaxillary edentulism and requiring removable dentures. Exclusion criteria were defined as follows: anatomical deformities of the external ear and nose, ridge deformities, oral diseases (such as oral submucous fibrosis and temporomandibular disorders), and systemic diseases affecting neuromuscular function (e.g., neuromuscular disorders and osteoporosis).

### 2.3. Predictor and Outcome Variables

The primary predictor variable was the placement of complete removable dentures. Outcome variables included changes in cephalometric landmarks, cranio-cervical posture, and nasopharyngeal airway dimensions measured through cephalometric analysis.

### 2.4. Data Collection and Measurement

Clinical and cephalometric evaluations were performed before and after prosthetic rehabilitation. The cephalometric measurements were conducted according to the Tweed and Rocabado guidelines. Standard cranial lateral X-rays were obtained with patients in a standardized upright position, with the Frankfort horizontal plane parallel to the ground and the natural head posture was maintained. Breathing was calm, without swallowing during imaging.

Key anatomical points measured included those listed in Table 1.

### 2.5. Statistical Analysis

Statistical analysis was conducted using SPSS software (version 15.00, SPSS, Chicago, IL, USA). Data normality was assessed using the Shapiro–Wilk test. Quantitative data were expressed as means ± standard deviations. A paired samples t-test was used for pre- and post-treatment comparisons, considering a bilateral *p*-value of <0.05 statistically significant.

## 3. Results

### 3.1. Demographic Data

A total of 12 participants (5 males and 7 females; mean age 65 ± 5.2 years, range 55–75) were included in this pilot study. The distribution of skeletal typology was as follows: four hyperdivergent, two normodivergent, and six hypodivergent patients (Table 2).

### 3.2. Overview of Cephalometric Changes

**Figure 1:**
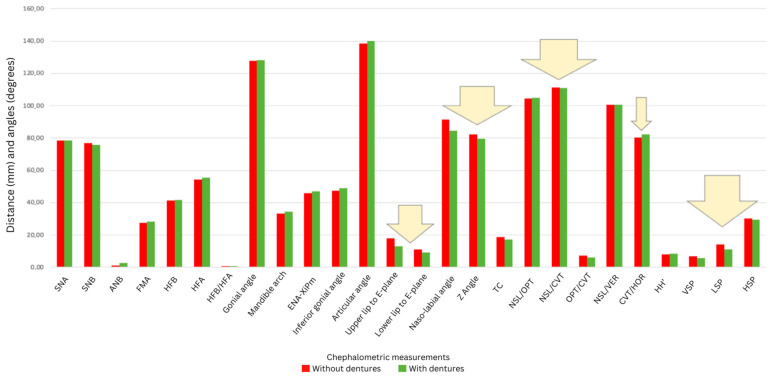
Comparison of facial profile changes before and after denture placement (arrows indicate statistically significant changes).

All measured cephalometric variables demonstrated modifications following prosthetic rehabilitation. Significant common findings included an advancement of the upper lip relative to the aesthetic plane (by 4.74 mm, *p* = 0.004) and a reduction in the anteroposterior diameter of the oropharyngeal lumen (from 12.75 mm to 10.27 mm, −19.5%, *p* = 0.035) (Figure 1). The results also indicated an advancement of the upper lip by 4.74 mm compared to the aesthetic plane (plane passing from Pn to Pog’, *p* = 0.004) and by 2.84 mm for the lower lip (*p* = 0.176) for complete denture wearers. A decrease was observed for both VPS (approx. 17.7%, *p* = 0.076) and LPS (approx. 2.2%, *p* = 0.071) after the placement of the complete dentures. A statistically significant difference in the anteroposterior diameter of the oropharyngeal lumen, with and without bimaxillary complete dentures, was identified, as a decrease from 12.75 mm to 10.27 mm, or approximately −19.5%, was observed (*p* = 0.035).

Figure 1 illustrates the average anterior displacement of the lips relative to the aesthetic plane (line passing through points Pronasale to soft tissue Pogonion). Arrows indicate statistically significant shifts, highlighting the clinical impact of prosthetic treatment on soft tissue profile.

**Figure 2:**
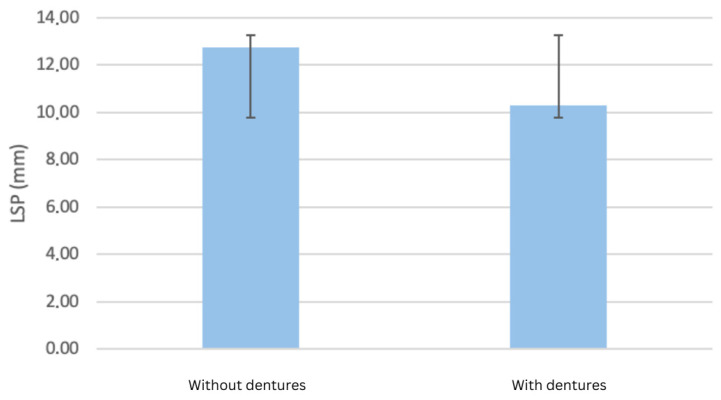
Changes in oropharyngeal airway dimensions (LPS) measured using cephalometric analysis.

Figure 2 shows a significant reduction of approximately 19.5% in the anteroposterior diameter of the oropharynx after complete denture placement. This reduction may reflect decreased airway patency and altered tongue positioning.

### 3.3. Cranio-Cervical Posture Changes

**Figure 3:**
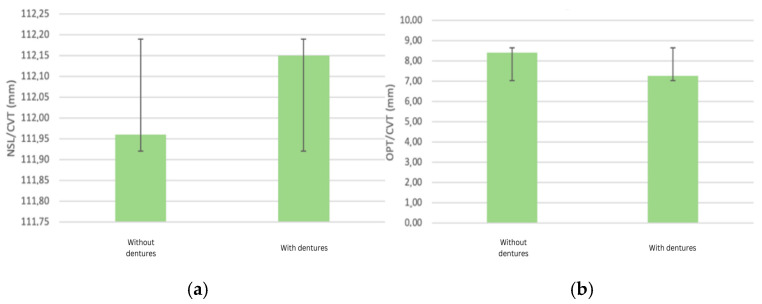
(**a**) Alterations in cervical lordosis (NSLT/CVT angle) after denture placement; (**b**) alterations in cervical lordosis (OPT/CVT angle) after denture placement.

When analyzing the changes in the inclination of the cranial base, the corresponding angles exhibited an increase, indicating cephalic extension. Specifically, the NSL/CVT angle showed a slight increase from 111.96° to 112.15° after the placement of the complete denture (*p* = 0.90). The OPT/CVT angle showed a decrease after the placement of complete dentures from 8.42° to 7.28°, representing an average attenuation of lordotic curvature of −13.5% when wearing the prosthesis (*p* = 0.21). A slight increase indicates a trend towards cephalic extension, suggesting compensatory postural adjustments due to changes in vertical dimension and mandibular positioning (Figure 3a). The observed decrease suggests attenuation of cervical lordosis, potentially due to improved mandibular positioning and altered muscular balance post-rehabilitation (Figure 3b).

### 3.4. Skeletal Typology-Dependent Changes

The results of measurements based on the different skeletal typologies are displayed in Table 3. In the case of hyperdivergent patients, the treatment goal was to achieve a reduction in the vertical dimension of the lower face after the placement of the dentures; the results indicated a decrease in the FMA angle and a reduction in the anterior facial height (−1.95 mm). The vertical index HFP/HFA showed compensation for the skeletal pattern (+0.02). For normodivergent patients (FMA = 22–28), the treatment aimed to maintain a normal vertical dimension of the lower face while restoring aesthetic and functional harmony. In the case of the FMA angle, a slight reduction was observed (−0.2 degrees); however, it remained within the normal range of values. The vertical index HFP/HFA showed no modification, remaining at 0.710, while ENA-Xi-Pm indicated a slight increase. No modification in the HH’ distance was observed in normodivergent patients undergoing minimal changes in VDO. For hypodivergent patients (FMA < 22), the prosthetic treatment achieved a correction of the FMA angle in relation to the patient’s typology towards a normal range. The results of the SNB angle indicated a posterior rotation of the mandible in its hinge axis with a slight increase in vertical dimension. An increase in the lower gonial angle (+4.43 degrees) was observed after prosthesis placement. Meanwhile, there was a decrease in the mandibular arch. A decrease in the HH’ distance (−1.7 mm) was observed following an increase in VDO in hypodivergent patients.

**Figure 4:**
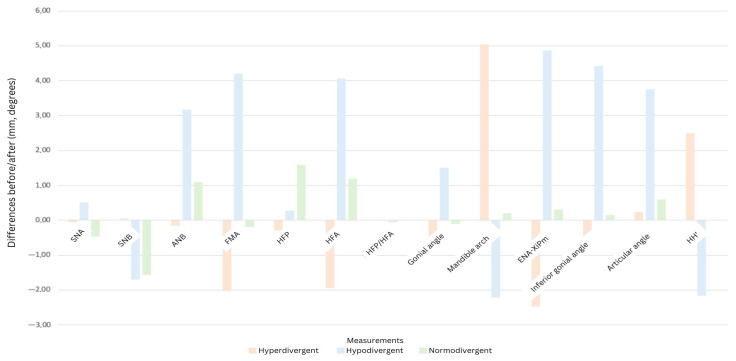
Histogram representing the changes obtained according to skeletal typologies.

Figure 4 illustrates these variations across skeletal typology, allowing the observation of the variability and direction towards which complete dentures can compensate the skeletal typology.

Patients were divided into three groups based on skeletal typology to assess how complete dentures compensated differently:

Hyperdivergent patients showed decreased vertical dimension of the lower face, decreased FMA angle, and anterior facial height reduction (−1.95 mm). The vertical index HFP/HFA showed slight compensation (+0.02).

In normodivergent patients, the aim was to maintain normal vertical dimension and aesthetic harmony. Minor changes included a slight reduction in the FMA angle (−0.2°) while ENA-Xi-Pm showed a slight increase.

In hypodivergent patients, the prosthetic treatment achieved correction of the FMA angle to normal range with posterior rotation of the mandible and a slight increase in the vertical dimension. An increase in the lower gonial angle (+4.43°) was observed along with a decrease in the HH’ distance (−1.7 mm).

### 3.5. Limitations

The study’s limited sample size was primarily due to the stringent inclusion and exclusion criteria necessary to ensure participant homogeneity. Additionally, logistical constraints, such as patient availability and compliance with follow-up appointments, as well as the resources required for cephalometric procedures, contributed to the reduced number of participants. These factors reflect the feasibility challenges encountered during the study period. Therefore, the study’s findings should be interpreted cautiously, as they may be difficult to generalize.

## 4. Discussion

The aim of this pilot study was to address the following question: How does complete denture rehabilitation affect anatomical landmarks, cranio-cervical posture, and respiratory dimensions? We aimed to clarify the compensatory biomechanical and postural changes that occur with complete denture use. We hypothesized that wearing dentures would significantly impact these parameters. The null hypothesis was validated, and an important possible link was identified between body posture and prosthetic treatment. Our findings align with the existing literature and diverge from it in some features, underscoring the complexity of the impact of dental prosthetics on human anatomy.

Our findings generally align with the existing literature, which underscores the importance of dental occlusion in influencing cranio-cervical posture and respiratory function. The cranio-cervical posture alterations observed in our study support the biomechanical model proposed by Michelotti et al. [8], which emphasizes the interdependence of dental occlusion and cranio-cervical dynamics. The results of our pilot study revealed changes in facial soft tissues and airway space dimensions. These findings support the biomechanical models proposed by Manny et al. [9], Watanabe et al. [10], and Fujinami et al. [11]. Our results also reinforce the idea that wearing dentures impacts occlusal relationships and postural control mechanisms. Our observations suggest that cervical alignment and functional postural adaptations play critical roles in the rehabilitation process [11,12].

Despite the insights gained, the small cohort size of our pilot study limits generalizability. The low number of participants resulted from the strict inclusion criteria, logistical constraints, and the feasibility challenges of cephalometric imaging [13,14]. Nevertheless, the study’s strength lies in its prospective design, standardized cephalometric evaluation, and detailed analysis of skeletal typology-based differences [15,16,17].

The anatomy of the upper airway is modified during sleep in complete edentulous patients due to changes in the postural rest position of the mandible, tongue, and muscle tone [17]. In addition, similarities with a study conducted by Hasan S.G. [16] were identified, showing obstructive changes in the dimensions of the upper airways, as well as significant modifications in the dynamic measurements of the normal head position following the placement of complete dentures [16]. The modifications observed in our study align with those in Hasan S.G.’s research. A statistically significant difference in the narrowing of the oropharynx at its narrowest part between the posterior wall of the pharynx and the base of the tongue (LSP) was observed.

These findings highlight the need for individualized denture fitting, tailored to each patient’s skeletal pattern and craniofacial morphology, as proven in previous published studies [18,19,20]. The consequences of complete edentulism are represented by the decreased occlusal vertical dimension, a reduction in the lower facial height, and rotation of the mandible with a reduction in the airway space, and/or the hypotonicity of the pharyngeal musculature with increased airway resistance and aggravation of obstructive sleep apnea [20,21]. It is important to recognize the potential impacts of denture wear on posture and airway dimensions, particularly in hyperdivergent and hypodivergent patients who exhibited significant compensatory changes [19,22].

The modifications for the NSL/CVT and NSL/OPT angles represent cephalic flexion/extension, indicating either clockwise or counterclockwise rotation, respectively. Without ensuring a natural head position, it is only possible to analyze cervical posture, i.e., the position of the head relative to the cervical spine, based on these variables. This study did not analyze the NSL/- VER or CVT/HOR angles. For these angles, an increase corresponding either to cervical flexion if NSL is fixed, or to cephalic extension if the cervical spine is fixed, was identified. NSL remains stable when an inclination of the cranial base is imposed, for example, using a cephalostat. However, the cervical spine is considered to remain immobile in a natural head position [23].

Given the significant impact of complete dentures on cranio-cervical alignment and respiratory dimensions, it is imperative for dental practitioners to consider these factors during the fitting process [24,25]. Assessing changes in head posture and conducting follow-up evaluations can help optimize the functionality and comfort of dentures. Additionally, personalized adjustments based on the specific anatomical characteristics of each patient are recommended to enhance the overall effectiveness of the prosthetic rehabilitation.

Within the limitations of this study, we have found statistically significant differences regarding the upper lip position and in the narrowing of the oropharynx at its narrowest part.

A decrease in pharyngeal dimensions induced by a more backward position of the tongue base due to a decrease in the intraoral space occupied by the prosthesis is considered normal. However, it is important to consider the space necessary for proper respiration in order not to exacerbate existing ventilation problems.

Regarding head position, a modification in the OPT/CVT angle was identified, representing an attenuation of the cervical lordosis curvature by −13.5% during denture wear. However, no statistically significant difference could be established. It can be considered that this change is mainly due to the modification of 1.32 degrees in the NSL/OPT angle, resulting from cervical flexion if NSL is considered fixed. This modification is reflected in the OPT/CVT angle with a decrease of −1.13 degrees. Additionally, the results regarding the modification of the airways and head position seem to be consistent with research conducted by Hasan S.G [16], as well as research by Zhang [25].

One of the objectives during the design of complete removable dentures is to restore facial harmony, particularly the lower facial height. To achieve this, the anatomical characteristics of each patient must be considered to adapt the prostheses as closely as possible to their morphology [19]. For this reason, patients were divided into three categories according to their skeletal typology. This allowed us to see how the modifications depend on the patient’s morphology.

For hyperdivergent patients, the modifications in the position of anatomical features on cephalometry had a slight compensatory effect on skeletal typology by slightly reducing the VDO. In contrast, for hypodivergent patients, the modifications in the position of anatomical landmarks also had a compensatory effect on skeletal typology following an increase in VDO. Finally, for normodivergent patients, the modifications in the position of anatomical landmarks varied minimally, indicating no compensation of skeletal typology and, thus, few modifications in the measurements.

Correlations in angle changes following VDO modification were highlighted, based on the table created by JD Orthlieb et al. [17]. This facilitated the observation of the improvement ensured by the complete dentures on the correlation of angles, namely the mandibular arch, inferior gonial angle, and ENA-Xi-pm. Additionally, the ENA-Xi-pm value lies outside the range proposed between the mandibular arch and the inferior gonial angle. This is due to the compensation of the skeletal typology, which is why the obtained ENA-Xi-pm angle was below this range for hyperdivergent patients (decreased ENA-Xi-pm), and above it for hypodivergent patients (increased ENA-Xi-pm).

In this study, some of the modifications in cephalometric parameters before and after complete denture placement were statistically significant. However, these results should be interpreted with caution because of the limited sample size. Larger and more homogenous samples may be needed to confirm the current results. Future studies should focus on larger, multicentric research to confirm these findings across diverse populations. Variation in residual alveolar ridge reduction, tongue characteristics in terms of volume and pressure, and electromyographic activity of the head and neck muscles after the placement of a complete denture, are required to analyze the detailed nature of the mechanisms involved in the process of edentulous patients’ rehabilitation. A study by Suzuki [26] followed the changes in the tongue movement, maintaining the normal form of the tongue during MRI investigations, with stable swallowing associated with occlusal reconstruction.

Recent advancements in mandibular motion tracking offer promising tools for understanding the functional consequences of edentulism. Ruggiero et al. [27] demonstrated that open-source optical jaw tracking systems can capture detailed mandibular dynamics, providing clinicians with precise, reproducible information on jaw movement patterns that exceed the capabilities of traditional articulators or static records. These tools are particularly valuable in edentulous patients, whose altered occlusal support and neuromuscular control often lead to compensatory mandibular movements and postural adaptations. Building on this research, Gismondi et al. [28] proposed a fully digital workflow that combines intraoral scanning with optical axiography to fabricate diagnostic occlusal devices. A functional “utility position” derived from natural mandibular movements has been identified, and these data have been imported into CAD software. This technique allows for the design of prosthetic devices that are personalized to each patient’s actual functional envelope. The integration of such motion-based data into prosthetic rehabilitation represents a significant shift from static to dynamic planning and may offer a means of minimizing the postural and airway-related alterations associated with edentulism. Applying these approaches could enhance diagnostic accuracy, improve patient comfort, and contribute to more physiologically adapted rehabilitations.

### 4.1. Limitations of This Study

The small cohort size and observational nature of this pilot study limit the generalizability of the findings. Other limitations include short follow-up period and the inability to measure certain parameters, such as residual ridge resorption and tongue posture. Nevertheless, the prospective design and standardized methodology provide valuable preliminary insights. Future larger studies are needed to confirm these results.

### 4.2. Future Research Perspectives

Further research in this area could improve the quality of life for patients undergoing bimaxillary prosthetic rehabilitation and enable clinicians to make evidence-based treatment decisions. While our findings contribute valuable knowledge, caution is warranted in generalizing them due to the limited sample size and observational study design. Future interventional studies should examine the long-term outcomes of various denture adjustment protocols to establish best practices in prosthetic rehabilitation and ensure more predictable, patient-specific outcomes.

## 5. Conclusions

This pilot study demonstrated that complete dentures treatment might determine compensatory changes in anatomical landmarks, cranio-cervical posture, and nasopharyngeal airway dimensions. These modifications vary according to skeletal typology, which underscores the importance of personalized prosthetic treatment planning. Our findings emphasize the potential of complete dentures to influence respiratory function by reducing oropharyngeal lumen dimensions and postural alignment by promoting cephalic extension and attenuating cervical lordosis. Despite these promising insights, further confirmation through larger, multicentric studies is warranted.

## Figures and Tables

**Table 1:** Abbreviations and descriptions of cephalometric landmarks and measurements.

Abbreviation	Full Description
NSL	Nasion-sella line (cranial base reference line)
CVT	Cervical vertebra tangent (line tangent to cervical vertebrae curvature)
OPT	Odontoid process tangent (line tangent to odontoid process curvature)
VDO	Vertical dimension of occlusion
VPS	Velopharyngeal space (nasopharynx)
LPS	Lingual pharyngeal space (oropharynx)
HPS	Hypopharyngeal space (laryngopharynx)
FMA	Frankfort–mandibular plane angle
HFP/HFA	Height facial posterior/height facial anterior ratio
ENA-Xi-Pm	Angle formed by anterior nasal spine, Xi point, and protuberance mentis
HH’	Distance between hyoid bone and mandibular plane
SNB	Angle formed by the sella-nasion and nasion-B point
Pn	Pronasale
Pog’	Soft tissue pogonion

Nasopharyngeal space (VPS); oropharyngeal space (LPS); laryngopharyngeal space (HPS); angles: NSL/CVT, OPT/CVT; vertical dimension of occlusion (VDO); cephalometric analysis did not include artificial teeth or prosthetic thicknesses due to visibility constraints. An error margin of ±0.5 degrees/mm was accepted.

**Table 2:** Demographic data of the participants.

Characteristic	Value
Number of participants	12
Age (mean ± SD)	65 ± 5.2 years (range: 55–75)
Gender	Male: 5 (41.7%), Female: 7 (58.3%)
Skeletal typology	Hyperdivergent: 4 (33.3%) Normodivergent: 2 (16.7%) Hypodivergent: 6 (50.0%)
Follow-up period	6 months (post-treatment evaluation)

**Table 3:** Cephalometric measurements before and after denture placement across skeletal typologies.

Measurement	Hyperdivergent Without	Hyperdivergent With	Hypodivergent Without	Hypodivergent With	Normodivergent Without	Normodivergent With	Difference Hyper.	Difference Hypo.	Difference Normo.
SNA	77.98	77.93	78.59	79.10	78.15	77.68	−0.05	1.70	−0.47
SNB	75.16	75.21	75.37	73.67	79.70	78.14	0.05	−1.70	−1.56
ANB	2.83	2.67	2.22	5.39	−1.60	−0.50	−0.16	3.17	1.10
FMA	34.35	32.33	30.23	24.83	27.60	27.40	−2.02	−5.40	−0.20
HFP	40.55	40.55	43.97	44.23	39.40	41.00	0.00	0.27	1.60
HFA	57.75	55.80	49.80	53.87	55.80	57.00	−1.95	4.07	1.20
HFP/HFA	0.71	0.73	0.88	0.82	0.71	0.73	0.02	−0.06	0.02
Gonial angle	133.61	133.11	120.33	121.74	129.40	129.30	−0.50	1.51	−0.10
Mandibular arch	26.05	31.09	38.66	36.43	35.40	35.50	5.04	−2.22	0.20
ENA-XiPm	52.89	50.42	40.76	45.63	45.63	44.50	−2.47	4.87	−1.13
Lower gonial angle	51.38	50.75	44.35	48.78	46.90	47.05	−0.63	4.43	0.15
Articular angle	137.18	137.42	138.73	142.48	139.10	139.70	0.24	3.75	0.60
Upper lip to E-plane	18.87	15.17	15.26	12.20	19.80	13.88	−3.70	−3.06	−5.92
Lower lip to E-plane	9.66	10.15	13.80	8.22	8.22	8.90	0.49	−5.58	−1.30
Nasolabial angle	99.96	88.13	91.21	100.90	83.50	65.00	−11.83	9.69	−18.50
Angle Z	84.20	86.25	82.86	74.49	78.00	78.20	2.05	−8.37	−2.20
TC	23.30	21.91	16.73	14.77	15.70	15.20	−1.40	−1.97	−0.50
NSL/OPT	105.88	105.70	101.20	104.30	106.00	105.00	−0.18	3.10	−1.00
NSL/CVT	118.13	114.95	109.97	111.73	106.50	106.00	−3.18	2.07	−0.50
OPT/CVT	12.30	9.35	8.47	7.43	10.50	9.00	−2.95	−1.03	−1.50
NSL/VER	100.18	100.10	101.07	101.87	99.50	99.50	−0.08	0.80	0.00
CVT/HOR	72.90	75.20	85.67	85.03	82.60	86.50	2.30	−0.64	3.90
HH’	9.00	11.50	5.00	2.83	5.10	3.10	2.50	−2.17	−1.90
VSP	4.30	4.40	4.70	5.80	8.40	7.10	0.10	1.50	−1.30
LSP	11.95	10.10	10.83	9.23	20.10	13.70	−1.85	−1.60	−6.40
HSP	33.00	31.79	26.20	26.00	31.70	30.80	−1.22	−0.20	−0.90

## Data Availability

The raw data supporting the conclusions of this article will be made available by the authors on request.
